# Converting sounds to meaning with ventral semantic language networks: integration of interdisciplinary data on brain connectivity, direct electrical stimulation and clinical disconnection syndromes

**DOI:** 10.1007/s00429-021-02438-x

**Published:** 2022-03-10

**Authors:** Viktoria Sefcikova, Juliana K. Sporrer, Parikshit Juvekar, Alexandra Golby, George Samandouras

**Affiliations:** 1 UCL Queen Square Institute of Neurology, University College London, London, UK; 2 Department of Neurosurgery, Brigham and Women’s Hospital, Harvard Medical School, Boston, MA USA; 3 Department of Radiology, Brigham and Women’s Hospital, Harvard Medical School, Boston, MA USA; 4 Victor Horsley Department of Neurosurgery, The National Hospital for Neurology and Neurosurgery, London, UK

**Keywords:** Ventral stream, Semantics, Language, Brain networks, White matter

## Abstract

Numerous traditional linguistic theories propose that semantic language pathways convert sounds to meaningful concepts, generating interpretations ranging from simple object descriptions to communicating complex, analytical thinking. Although the dual-stream model of Hickok and Poeppel is widely employed, proposing a dorsal stream, mapping speech sounds to articulatory/phonological networks, and a ventral stream, mapping speech sounds to semantic representations, other language models have been proposed. Indeed, despite seemingly congruent models of semantic language pathways, research outputs from varied specialisms contain only partially congruent data, secondary to the diversity of applied disciplines, ranging from fibre dissection, tract tracing, and functional neuroimaging to neuropsychiatry, stroke neurology, and intraoperative direct electrical stimulation. The current review presents a comprehensive, interdisciplinary synthesis of the ventral, semantic connectivity pathways consisting of the uncinate, middle longitudinal, inferior longitudinal, and inferior fronto-occipital fasciculi, with special reference to areas of controversies or consensus. This is achieved by describing, for each tract, historical concept evolution, terminations, lateralisation, and segmentation models. Clinical implications are presented in three forms: (a) functional considerations derived from normal subject investigations, (b) outputs of direct electrical stimulation during awake brain surgery, and (c) results of disconnection syndromes following disease-related lesioning. The current review unifies interpretation of related specialisms and serves as a framework/thinking model for additional research on language data acquisition and integration.

## Introduction to language models

Despite the accumulated theoretical and clinical evidence over the last two centuries, the anatomical basis of semantic (*Gr. σημασία, meaning*) neural networks, converting sounds to meaning, remains incomplete. The current, large-scale, anatomo-functional model of language employed in brain mapping during tumour resection is largely based on the dual-stream model proposed by Hickok and Poeppel (2000, 2004, 2007). The model includes a dorsal stream, mapping the sounds of acoustic speech to articulatory, motor, and phonological networks, and a ventral stream, mapping the sounds of acoustic speech into meaningful, conceptual, and semantic representations (Hickok and Poeppel 2000). The Hickok–Poeppel model was conceptually based on the 1992 visual dual-stream model of Milner and Goodale with a ventral or “what” stream, identifying objects, and a dorsal or “how” stream, guiding movement, reaching and grasping with visual objects (Goodale and Milner 1992).

The dual-stream language model is not novel, neuroanatomically, as it had been proposed by Wernicke in 1874, and later by Lichtheim in 1884, with an auditory (sensory) and a motor component (Anderson et al. 1999). Influenced by his teacher Meyenert, Carl Wernicke postulated the disconnection of the posterior temporal region, the seat of “word images” or historically termed “Wernicke’s area”, from the anterior motor programming regions would result in impaired spontaneous speech, naming, and repetition (Anderson et al. 1999). Wernicke went beyond the localization of function in brain areas and started building models of association between cerebral regions for the elaboration of language functions, typically conduction aphasia (Anderson et al. 1999). The concept of disconnection was further developed by Lichtheim, adding transcortical and subcortical motor aphasia, and transcortical and subcortical sensory aphasia to the range of language function subsequent to a brain disconnection-associationist model (Anderson et al. 1999; Krestel et al. 2013).

Following Wernicke, Dejerine highlighted the anterior inferior frontal area, historically termed “Broca’s area”, could still produce aphasia through subcortical fibre damage and Pierre Marie further characterized the zone of white matter beneath the surface of Broca’s area responsible for aphasia (Krestel et al. 2013). Geschwind postulated in 1965 and 1970 that inferior parietal lobe lesioning indirectly disconnects historically termed Broca’s to Wernicke’s area exchanges and also results in conduction aphasia, introducing the traditional Wernicke–Lichtheim–Geschwind language model (Anderson et al. 1999). However, neuroclinicans and neurosurgeons have long recognized language is not restricted to the anterior inferior frontal and posterior temporal cortices, as resection of these regions does not invariably cause deficits and conversely, deficits may occur when resecting other cortical or subcortical segments, even if these areas and the arcuate fasciculus remain intact (Plaza et al. 2009; Lubrano et al. 2010).

However, despite the lengthy evolution of language models and accumulated evidence, there is still widespread lack of consensus with regard to anatomical definitions, presumed function, and models of testing of most white matter tracts (Dick and Tremblay 2012). Indeed, high variability in white matter mapping paradigms, selection and administration of intraoperative tasks, semantic error rates, and termination points for surgery is extensively reported (Duffau et al. 2002; Ruis 2018; Sefcikova et al. 2020).

These observations are of significant interest to neuroclinicians and neurosurgeons, as understanding neurological syndromes or resecting white matter tracts affected by tumours requires comprehension of the function of each pathway, so appropriate intraoperative tasks can be selected and interpreted. Indeed, large-scale studies have shown that when appropriate intraoperative testing is applied with awake brain mapping (ABM), not only larger tumour areas can be resected but also neurological deficits are substantially minimised. A meta-analysis and systematic review of 90 observational studies, examining the role of ABM in surgery for supratentorial gliomas, found gross total resection in patient cohorts with and without ABM were 75% and 58%, respectively, while persistent neurological deficits were recorded in 3.4% and 8.2% of cases, respectively (De Hamer et al. 2012).

However, the implication of currently recorded controversies affect the wider neuroscience community, as white matter tracts’ anatomy and function are studied separately, and often in isolation, with diverse techniques including postmortem blunt fibre dissection, histochemical tract-tracing techniques, diffusion MRI (dMRI) methods, and intraoperative ABM.

The current review aims to present a comprehensive, interdisciplinary synthesis of ventral, semantic connectivity pathways consisting of the uncinate, middle longitudinal, inferior longitudinal, and inferior fronto-occipital fasciculi, with special reference to areas of controversies or consensus. The inclusion of four, distinct white matter tracts of the ventral pathway system was based on the prevailing model of Hickok and Poeppel, as a method of white matter pathway segregation, although the data reviewed are not aimed at validating any particular language model. Importantly, the Hickok–Poeppel model, based on auditory processing of speech, would not be applicable to all components of semantic processing, including reading and writing. The aim of the current review is not to validate specific language models, but rather to provide neuroclinicans with a theoretical and practical background, so lesioning syndromes from acute brain injury or stroke, and intraoperative direct electrical stimulation results can be better understood. Conversely, basic neuroscientists may find clinical results of relevance to validate existing, or initiate new, theoretical models.

To address a comprehensive, interdisciplinary synthesis, this review is based on critically analysed data from various disciplines, ranging from tract-tracing photomicrography to advanced imaging and neuropsychiatry. Each method carries limitations, but integrating data outputs facilitates our current understanding (Dick and Tremblay 2012). For each tract, the same structured template was adopted, consisting of brief historical evolution of conceptual models; anatomical, fibre organisation models; functional considerations derived from applied neuroscience and clinical neurology, resulting from progressive changes in white matter microarchitecture, such as in schizophrenia, suggesting a long-term disruption of functional connectivity; and finally, data from dissociation syndromes, either temporary, stimulation-induced, or permanent, secondary to irreversible injury, such as brain injury or stroke. The heterogeneity of pathway functions often expand beyond language streams, and therefore, such references were included for completeness. Unless explicitly stated, functions discussed refer to the left hemisphere. To the authors’ best knowledge, a synthetic, analytical review integrating seemingly diverse disciplines has not been previously published.

The review is supplemented by representative dissections of white mater pathways. Using a modified Klinger technique, formalin-fixed cadaveric brains were subsequently frozen at − 10 to – 15 °C for 10–14 days and then thawed and dissected under a Zeiss operating microscope (Zeiss, Oberkochen, Germany) with use of micro-instruments and wooden spatulas. Institutional permission for dissections was obtained and photographs were taken from identifiable and electronically tagged specimens, according to Human Tissue Act, 2004, HAU best practice guidelines and the Data Protection Act, 1998. Authorization was obtained from the Human Anatomy Unit, Division of Surgery, Imperial College London, London, UK. Structures were sequentially identified and are described below, with accompanying photographs.

## Uncinate fasciculus (UF)

### History of the UF

Initially described by Reil (1809) as a fibre system connecting anterior frontal and middle temporal lobes, the UF was subsequently defined by Burdach (1822) as related to the lentiform nucleus and external capsule, a model that persisted throughout the nineteenth century. The UF was considered the shortest of five major association bundles by Dejerine, who distinguished medial from laterally placed fibres (Dejerine 1895).

### Fibre anatomy and models of the UF

#### Rostral and ventral terminations

**Fig. 1:**
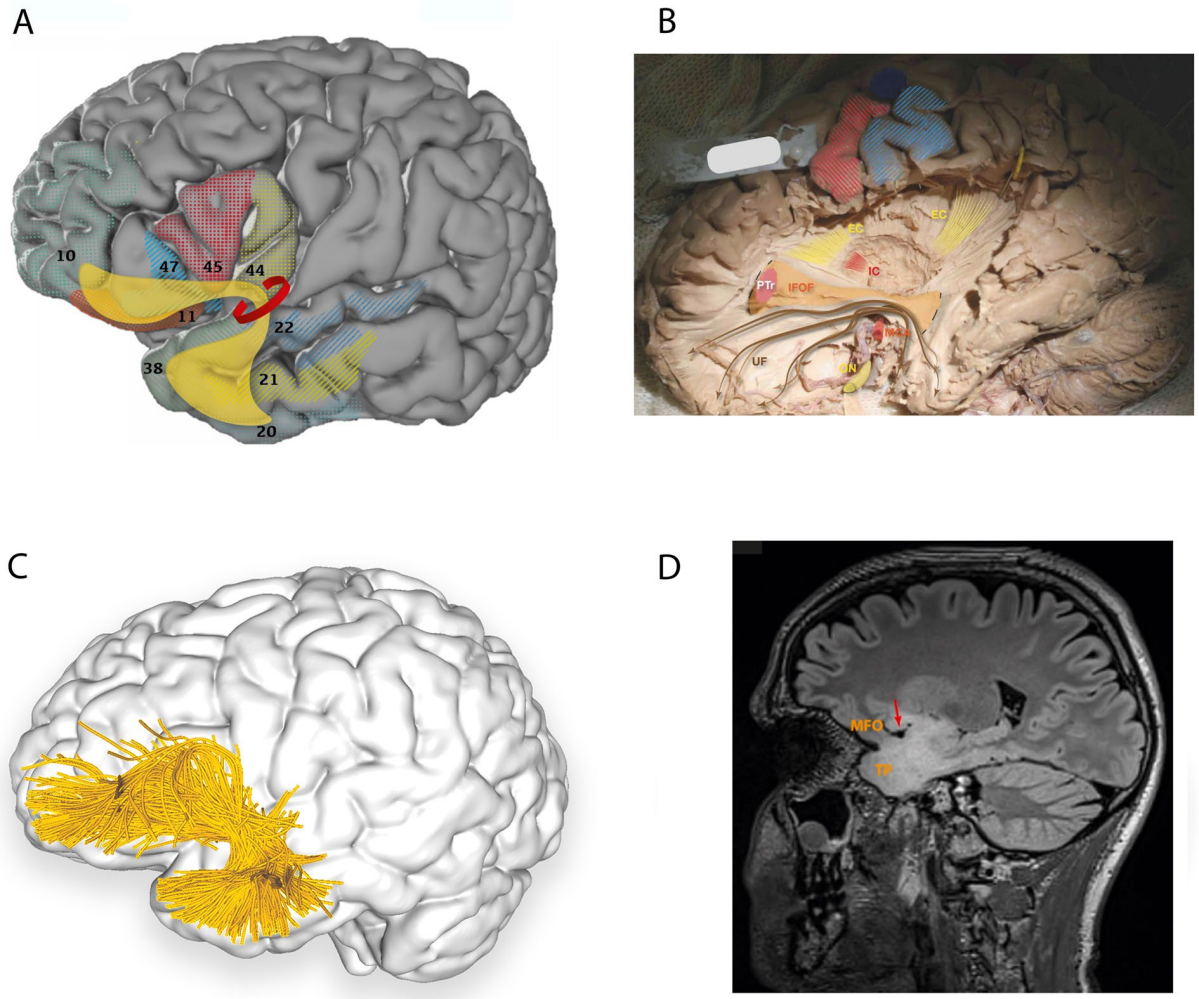
**A** Cortical projection of UF fibres (yellow) with numbered Brodmann areas which interconnect (grey background 3D model of brain from bigbrain.loris.ca; parcellation, numbering, overlays and all artwork from the authors using Adobe Illustrator, Creative Cloud 2020). **B** Original fibre dissection of the left UF on a hemisphere prepared with the Klinger technique. Note the hook-like arrangement of the UF around the MC. The IFOF is partially exposed (limits of exposure indicated by dotted lines) and its narrowest point corresponds to the narrowest point of the UF. Primary motor and sensory cortices are highlighted in red and blue, respectively. **C** Left lateral view of the brain superimposed with the left uncinate fasciculus (purple) derived by automated white matter tract parcellation using the white matter analysis software provided in SlicerDMRI (Norton et al. 2017; Zhang et al. 2018, 2020), applied to a single-subject dataset from the Human Connectome Project (Van Essen et al. 2013). This automated clustering and parcellation pipeline employs Unscented Kalman Filter (UKF) tractography (Malcolm et al. 2010) by seeding all voxels where fractional anisotropy (FA) is greater than 0.1. Tracking is stopped when FA falls below 0.08 or the normalized mean signal is less than 0.06. The minimum fibre length is set at 40 mm. In this figure, 30% of the total fibres comprising the tract are displayed and further refined by using multiple negative regions of interest (ROIs) to be reflective of a morphologically classical representation of the uncinate fasciculus. **D** Parasagittal FLAIR MRI scan of a high functioning 34-year-old patient who underwent routine, preoperative neuropsychological assessment, demonstrating significant underperformance (8/18) in the “naming of famous faces” task. Signal change, indicative of a low-grade glioma, demonstrates the involvement of the temporal pole and medial fronto-orbital gyri, connected by the UF typically arching around the middle cerebral artery (arrow). For orientation, please compare with middle cerebral artery location in **B**. The data in **B**–**D** are from three separate patients. *EC* external capsule (dorsal claustrocortical system), *IC* internal capsule, *IFOF* inferior fronto-occipital fasciclus, *MFO* medial fronto-orbital gyri, *MCA* middle cerebral artery, *PTr* pars triangularis, *TP* temporal pole, *ON* optic nerve, *UF* uncinate fasciculus

Methodologies, including blunt fibre dissections, diffusion MRI (dMRI) with single-region of interest (ROI), double-ROI, and/or multi-ROI, produce relatively consistent findings with rostral terminations including: (1) frontal operculum (Friederici et al. 2006) and (2) fronto-orbital cortex (Catani et al. 2002; Zhang et al. 2010; Thiebaut de Schotten et al. 2012; Hau et al. 2016). The ventral terminations, restricted to the temporal lobe, include: (1) anterior temporal lobe (ATL) (Catani et al. 2002; Friederici et al. 2006) and temporal pole (Catani et al. 2002; Thiebaut de Schotten et al. 2012; Hau et al. 2016), (2) superior temporal gyrus (Zhang et al. 2010; Hau et al. 2016), and (3) middle temporal gyrus (Zhang et al. 2010; Hau et al. 2016) (Fig. 1, Table 1). However, fibre variability and heterogeneity across subjects are commonly observed (Hau et al. 2016).

**Table 1:** Reported tract terminations are detailed for specific studies investigating the uncinate fasciculus, inferior longitudinal fasciculus, middle longitudinal fasciculus, and inferior fronto-occipital fasciculus

References	Tract terminations			
	Frontal	Temporal	Parietal	Occipital
Uncinate fasciculus				
Ebeling and Cramon (1992)	Rectus gyrus (BA 11), medial retro-orbital cortex (BA 12), sub-callosal area (BA 25)	Temporal lobe (BA 20, 38), amygdala (BA 28, 34, 36)	N/A	N/A
Catani et al. (2002)	Orbito and polar frontal cortex	ATL/TP	N/A	N/A
Friederici et al. (2006)	FOP (BA 44, 45, 47)	ATL (BA 38, 22)	N/A	N/A
Zhang et al. (2010)	SFG, medial orbitofrontal gyrus, lateral orbitofrontal gyrus	ITG, MTG, STG	N/A	N/A
Thiebaut de Schotten et al. (2012)	Orbitofrontal cortex (11, 47), frontal pole (BA 10), cingulate gyrus (BA 32)	TP (BA 38), uncus (BA 35), parahippocampal gyrus (BA 36, 30), amygdala	N/A	N/A
Hau et al. (2016)	MFG, IFG, lateral orbitofrontal gyrus, medial orbitofrontal gyrus, rectus gyrus	TP, STG, MTG, ITG, fusiform gyrus, entorhinal gyrus	N/A	N/A
Inferior longitudinal fasciculus				
Catani et al. (2002)	N/A	STG, MTG, ITG, fusiform gyrus	N/A	Lingual, cuneus, lateral occipital lobe, occipital pole
Catani et al. (2003)	N/A	Anterior lateral temporal cortex (BA 38), uncus/parahippocampal gyrus (BA 20, 36)	N/A	Dorsolateral occipital cortex (BA 18), posterior lingual/fusiform gyrus (BA 18, 19, 37)
Zhang et al. (2010)	N/A	STG, MTG, ITG	N/A	Lingual gyrus, fusiform area, SOG, MOG, IOG
Latini (2015)	N/A	Dorsolateral occipital ILF: TP (BA 38)	N/A	Dorsolateral occipital ILF: anterior MOG just posterior to human motion area (BA 19)
	N/A	Cuneal ILF: TP (BA 38) in subcortical MTG and ITG	N/A	Cuneal ILF: medial cuneal cortex (BA 19)
	N/A	Ventral ILF: TP (BA 38), subcortical ITG	N/A	Ventral ILF: posterior fusiform gyrus/basal occipital region (BA 19, 37)
	N/A	Li-Am ILF: medial temporal cortex (BA 21), parahippocampal/amygdaloid region (BA 28, 34)	N/A	Li-Am ILF: mesial posterior lingual cortex (BA 19)
Latini et al. (2017)	N/A	Fusiform ILF: STG (BA 22), MTG (BA 21), ITG (BA 20), parahippocampal gyrus (BA 34), fusiform (BA 37)	N/A	Fusiform ILF: Lateral occipital-temporal gyrus (BA 19)
	N/A	Dorsolateral occipital ILF: STG (BA 22), MTG (BA 21), ITG (BA 20), parahippocampal gyrus (BA 34), lateral occipital-temporal gyrus (BA 19, 37)	N/A	Dorsolateral occipital ILF: SOG, MOG, IOG (BA 18, 19)
	N/A	Lingual ILF: STG (BA 22), MTG (BA 21), ITG (BA 20), parahippocampal gyrus (BA 34), lateral occipital-temporal gyrus (BA 19, 37)	N/A	Lingual ILF: visual area 3/lingual gyrus (BA 19)
	N/A	Cuneal ILF: STG (BA 22), MTG (BA 21), ITG (BA 20), parahippocampal gyrus (BA 34), lateral occipital-temporal gyrus (BA 19, 37)	N/A	Cuneal ILF: cuneus (BA 19)
Middle longitudinal fasciculus				
Makris et al. (2009)	N/A	TP (BA 38), STG	AG (BA 39)	N/A
De Champfleur et al. (2013)	N/A	TP (BA 38), STG	AG (BA 39)	Lateral occipital area (BA 18, 19), occipital pole (BA 17, 18)
Makris et al. (2013|)	N/A	STG (BA 22, 42), TP (BA 38); temporal occipital region (BA 21, 37)	AG (BA 39), SMG (BA 40) SPL/precuneus (BA 7)	Occipital lobe (BA 18, 19), including the cuneus and lateral occipital area
Wang et al. (2013)	N/A	TP (BA 38), STG (BA 22)	SPL/ precuneus (BA 7), AG (BA 39)	Superior and middle occipital cortex/cuneus (BA 19)
Makris et al. (2017)	N/A	Dorsal TP (BA 38), STG (BA 22, 42)	AG (BA 39), SMG (BA 40), SPL/precuneus (BA 7)	Cuneus, lateral occipital area (BA 19, 18)
Kalyvas et al. (2020)	N/A	MLF-I: dorsolateral TP (BA 38), STG	MLF-I: SPL/precuneus (BA 7)	N/A
	N/A	MLF-II: dorsolateral TP (BA 38), STG	N/A	MLF-II: parieto-occipital area (BA 19)
	N/A	MLF-III: anterior TP and STS (BA 38)	N/A	MLF-III: superior or middle third of posterior occipital lobe/cuneus (BA 17, 18)
Inferior fronto-occipital fasciculus				
Catani et al. (2002)	Infero-lateral and dorsolateral frontal cortex	MTG, ITG, fusiform gyrus	Parietal cortex	Lingual gyrus
Lawes et al. (2008)	Segment I: lateral orbitofrontal gyrus (BA 47, 12)	N/A	N/A	Segment I: Inferior MOG (BA 18, 19)
	Segment II: frontomarginal gyrus (BA 10, 11)	N/A	N/A	Segment II: Inferior MOG (BA 18, 19)
	Segment III: frontomarginal gyrus (BA 10, 11)	N/A	N/A	Segment III: Lingual gyrus (BA 17, 18, 19)
	Segment IV: frontomarginal gyrus (BA 10, 11)	N/A	N/A	Segment IV: IOG (BA 17, 18, 19)
Martino et al. (2010)	Superficial/dorsal IFOF: frontal lobe	N/A	Superficial/dorsal IFOF: SPL (BA 7)	Superficial/dorsal IFOF: posterior SOG, posterior MOG (BA 18)
	Deep/ventral IFOF: frontal lobe	Deep/ventral IFOF: posterior temporobasal area (BA 37)	N/A	Deep/ventral IFOF: posterior IOG (BA 18)
Zhang et al. (2010)	Medial and lateral orbitofrontal gyrus, rectus gyrus, SFG, MFG, IFG	N/A	N/A	SOG, MOG, IOG
Thiebaut de Schotten et al. (2012)	Medial orbitofrontal cortex (BA 11), frontal pole (BA 10), SFG (BA 9)	N/A	N/A	Inferior and medial occipital lobe (BA 18, 19)
Sarubbo et al. (2013)	Superficial IFOF: pars triangularis (BA 45), pars orbitalis (BA 47)	Superficial IFOF: fusiform area (BA 37)	Superficial IFOF: SPL (BA 7)	Superficial IFOF: occipital extra-striate cortex (BA 18, 19)
	Posterior deep IFOF: MFG, DLPFC (BA 9, 46)	Posterior deep IFOF: fusiform area (BA 37)	Posterior deep IFOF: SPL (BA 7)	Posterior deep IFOF: occipital extra-striate cortex (BA 18, 19)
	Middle deep IFOF: MFG (BA 9, 10, 46), lateral orbitofrontal cortex (BA 12, 47)	N/A	Middle deep IFOF: SPL (BA 7)	N/A
	Anterior deep IFOF: frontal pole and basal orbitofrontal cortex (BA 10)	Anterior deep IFOF: fusiform area (BA 37)	N/A	Anterior deep IFOF: occipital extra-striate cortex (BA 18, 19)
Hau et al. (2016)	SFG, MFG, IFG, lateral orbitofrontal gyrus, medial orbitofrontal gyrus	STG, MTG, fusiform gyrus	AG, superior parietal gyrus	MOG, IOG, cuneus, lingual gyrus

Literature on the uncinate, inferior longitudinal, middle longitudinal, and inferior fronto-occiptial fascicles was accessed by PubMed, Web of Science, and Google Scholar, including material published until 2020. A combination of the following search terms were used: “dissection”, “diffusion tensor imaging”, “diffusion MR”, “diffusion magnetic resonance”, “diffusion MRI”, “uncinate”, “inferior longitudinal”, “middle longitudinal”, “inferior fronto-occipital”, “inferior fronto-occipital”, “fascicle”, and/or “fasciculus”. Studies were selected at the discretion of the authors and included studies that report terminations of at least one of the four ventral language tracts. Brodmann areas are approximations provided by the review authors

*AG* angular gyrus, *ATL* anterior temporal lobe, *BA* Brodmann area, *DLPFC* dorsolateral prefrontal cortex, *FOP* frontal operculum, *IFG* inferior frontal gyrus, *IFOF* inferior fronto-occipital fasciculus, *ILF* inferior longitudinal fasciculus, *IOG* inferior occipital gyrus, *ITG* inferior temporal gyrus, *MFG* middle frontal gyrus, *MLF* middle longitudinal fasciculus, *MOG* middle occipital gyrus, *MTG* middle temporal gyrus, *N/A* not available, *SFG* superior frontal gyrus, *SMG* supramarginal gyrus, *SOG* superior occipital gyrus, *SPL* superior parietal lobule, *STG* superior temporal gyrus, *STS* superior temporal sulcus, *TP* temporal pole, *UF* uncinate fasciculus

#### Lateralisation

Reports of both leftward and rightward lateralisation prompted analysis of specific UF components (Rodrigo et al. 2007; Hau et al. 2016; Ioannucci et al. 2020). Ioannucci and colleagues found the number of UF streamlines displayed significant rightward lateralisation (Ioannucci et al. 2020), whereas Hau and colleagues suggest right asymmetry in orbital and temporal branches (Hau et al. 2016), and Rodrigo and colleagues report sub-insular components are left lateralised (Rodrigo et al. 2007).

#### The three-segment model

The most common organisational structure of the UF involves three segments: (1) dorsal-temporal, (2) middle-insular, and (3) ventral-frontal (Ebeling and Cramon 1992). The ventral-frontal segment, based on dMRI of 74 healthy adults, was further subdivided into a classic frontopolar, lateral orbital, and a medial prefrontal branch terminating in the sub-genual cingulate gyrus (Bhatia et al. 2018). Early research described UF–cingulum connections, but now these two fibre bundles are considered distinct limbic association pathways, ventral and dorsal, respectively.

#### The two-fasciculi model

Initially, Catani et al. (2002) proposed that the UF is composed of two distinct fascicles: a dorsal/lateral originating at the frontal pole and a ventral/medial originating at the orbital cortex.

#### The three-stem model

Thiebaut de Schotten et al. (2012) further updated the UF segmentation into frontopolar, orbital, and temporal stems with the latter curving around the posterior insula. The updated Catani model supports that the two prefrontal stems are no longer distinct components at the temporal lobe.

### Functional considerations of the UF

The UF could serve as a point of communication between phonological and semantic networks. Performing a naming task includes processing facial or object stimuli by the visual system then selection of the relevant semantic representation, identification of the corresponding lexical representation (words), and finally identifying the phonological lexica, allowing for articulation. Disruption at various points of this processing pathway can explain the presence of both phonological and semantic disturbances upon UF stimulation or resection.

### Clinical functional implications: evidence from DES

#### Silent language impact

Surgical series report varied outputs following DES of the UF. In a series of 13 glioma patients, DES of the UF, employing counting and DO-80 picture naming, did not result in speech disturbances (Duffau et al. 2009). However, these findings could be explained by either the inferior fronto-occipital fasciculus (IFOF) providing functional compensation (Duffau et al. 2009, 2014) or failure of picture naming as a high-demand semantic function task.

#### Language deficits

In contrast, in a series of 44 patients, Papagno et al. (2011) reported phonemic paraphasias after temporal UF stimulation, and phonemic paraphasias followed by speech arrest after frontal UF stimulation. However, the limited stimulations applied over the UF during famous face and object naming precluded analysis of the tract’s role in naming. Although support for the role of the UF in language comes from a series of 36 awake craniotomies, stimulating cortical parcels commonly reported as left UF terminations identified 70 object-naming sites, 68 face-naming sites, and 42 overlapping cortical regions (Giussani et al. 2009).

### Clinical functional implications: evidence from disconnection syndromes

#### Famous face and object naming

Postoperative findings after UF resections provide further evidence for the UF’s role in famous face and object naming. However, this remains an area of controversy. Duffau et al. (2009) recorded short-term, reversible deficits only in naming tasks, following UF resection. In contrast, Papagno et al. (2011) found significant differences in picture naming at 3 months follow-up between patients with and without UF removal. Despite varying reaction cavities, postoperative deficits were worse in temporal UF and temporal pole resections, specifying UF segments necessary to preserve naming (Papagno et al. 2011). Significantly lower scores on postoperative object and famous face naming, and word list learning were reported (Papagno et al. 2011). A 9-month follow-up of 17 patients by the same group showed that while object naming and categorical verbal fluency recovered, famous face naming remained significantly impaired (Papagno et al. 2016), indicating possible implications in semantic memory preservation. Further support for the role of the UF in famous face naming comes from a 129-patient study, including patients with varying brain lesions, showing disruption of the UF is associated with impaired proper name retrieval (Mehta et al. 2016).

#### Semantic processing

The UF also appears to have a role in semantic processing generally, as evidence from primary progressive aphasia (PPA) shows UF tract-specific measures correlate with semantic processing scores, but not with verbal fluency measures (Catani et al. 2013). For the semantic variant of PPA, UF diffusivity changes correlate with difficulties on naming and single-word comprehension tasks (Catani et al. 2013). Further evidence comes from a series of 76 trauma-injured patients in which fractional anisotropy (FA) and percentage of lesion voxels in the UF significantly correlated with semantic task performance for both picture and sound naming, as well as picture-associative matching (a task specific for semantic processing) (Han et al. 2013). In a voxel-based morphometry analysis, comparing patients with semantic dementia—a condition characterized by progressive cognitive and language impairments, primarily related to semantic processing—to age-matched healthy controls, grey-matter atrophy in several UF terminations was reported, including the left inferior lateral temporal lobe, as well as the right temporal pole and ventromedial frontal cortex (Mummery et al. 2000). However, semantic memory only significantly correlated with left ATL atrophy (Mummery et al. 2000).

#### Behavioural changes

Different mechanisms of damage could explain the variations in behavioural pathology often noted for the UF. For example, microstructural changes in the UF bilaterally have been involved in antisocial behaviour (Waller et al. 2017) and conduct disorder (CD), featuring increased aggression and impulsivity (Zhang et al. 2014). When comparing studies investigating UF microstructural integrity in patients with PPA versus CD, reduced FA (in the left), as in PPA, correlates with semantic language deficits, while increased FA, as in CD (bilaterally), correlates with behavioural disturbance.

## Inferior longitudinal fasciculus (ILF)

### History of the ILF

Dejerine accurately described the ILF in 1895 as an associative system and included ILF in his “language zone” model (Dejerine 1895). Initial theories supported the ILF as a subcortical projection system of U-fibre series (Flechsig 1896), reinforced by Tusa and Ungerleider (1985) using both autoradiography and blunt dissection. Later, dMRI demonstrated the ILF as a direct occipito-temporal system (Catani et al. 2003), a model subsequently replicated by neuroimaging and dissection.

### Fibre anatomy and models of the ILF

#### Terminations and lateralisation

**Fig. 2:**
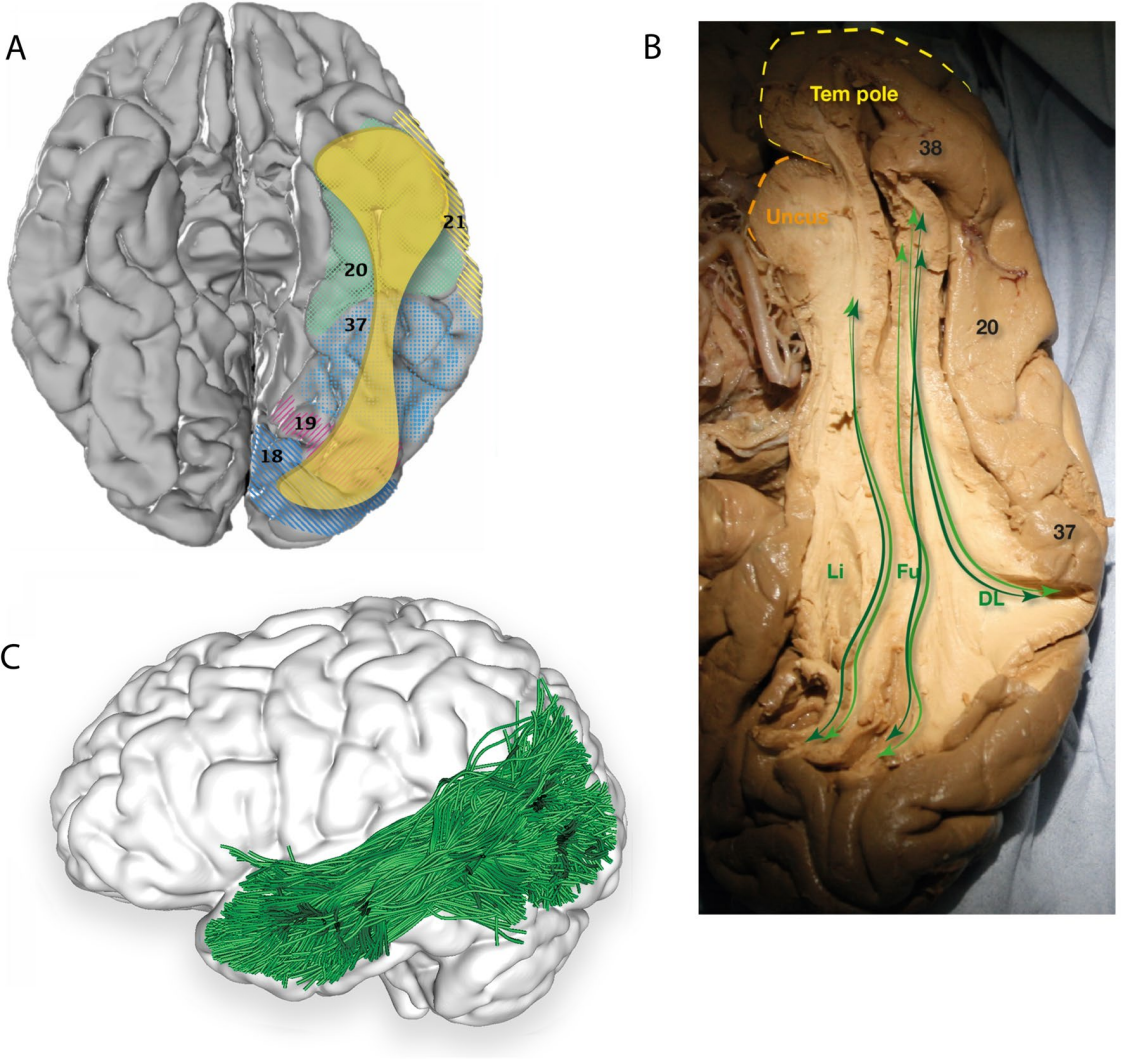
**A** Cortical projection of ILF fibres (yellow) in the basal surface of the left temporal lobe, created with the same methods as in Fig. 1A. **B** Original fibre dissection of the left ILF on a hemisphere prepared with the Klinger technique on the basal surface of the temporal hemisphere, demonstrating the dorsolateral, fusiform and lingual branches of the ILF. Numbers correspond to Brodmann areas. **C** Inferior view of the brain superimposed with the left inferior longitudinal fasciculus (blue) derived by automated white matter tract parcellation as in Fig. 1C. 20% of the total fibres comprising the tract have been displayed and further refined by using multiple negative regions of interest (ROIs) to be reflective of a morphologically classical representation of the inferior longitudinal fasciculus. *DL* dorsolateral, *Fu* fusiform, *Li* lingual, *Tem pole* temporal pole

Reported terminations of the ILF contain temporal and occipital regions, including the occipital cortex (Catani et al. 2002, 2003; Zhang et al. 2010; Latini 2015; Latini et al. 2017); temporal pole (Catani et al. 2003; Latini 2015); fusiform gyrus (Catani et al. 2002, 2003; Zhang et al. 2010; Latini 2015; Latini et al. 2017); parahippocampal gyrus (Catani et al. 2003; Latini 2015; Latini et al. 2017); amygdaloid area (Latini 2015); superior temporal gyrus (Catani et al. 2002; Zhang et al. 2010; Latini et al. 2017); middle temporal gyrus (Catani et al. 2002; Zhang et al. 2010; Latini et al. 2017); and inferior temporal gyrus (Catani et al. 2002; Zhang et al. 2010; Latini 2015; Latini et al. 2017) (Fig. 2, Table 1).

Behavioural findings suggest involvement of functional cortical regions, including the visual word form area (VWFA) (Mandonnet et al. 2007; Epelbaum et al. 2008; Duffau et al. 2014), visual object form area (VOFA) (Duffau et al. 2014), and fusiform face area (Hodgetts et al. 2015). In a dMRI analysis of 24 healthy subjects, Latani and colleagues found the volume of the ILF was significantly right-lateralised with no significant differences in ILF subcomponents (Latini et al. 2017).

#### The three-branch model

In 1962, Crosby and colleagues subdivided the ILF into lingual, cuneal, and lateral occipital branches, a model confirmed and expanded on by Catani et al. (2003) who virtually dissected the ILF in 11 healthy adults, confirming the three branches with four posterior origins: extra-striate cortex, cuneus, posterior lingual gyrus, and fusiform gyrus. The branches travel anteriorly and merge into a single tract at the posterior horn of the lateral ventricle, which terminates in the anterior lateral temporal cortex, parahippocampal gyrus, and amygdala (Catani et al. 2003).

#### The occipital termination model

Using postmortem dissection and dMRI, Latini identified three branches of the ILF’s main body, including: (1) the fusiform branch connecting the fusiform gyrus to the ATL, (2) the lingual branch connecting the lingual gyrus to the ATL, and (3) the dorsolateral occipital branch connecting the superior, middle, and inferior occipital gyri to the ATL (Latini et al. 2017). Removal of the dorsolateral occipital branch revealed a fourth, minor, cuneal branch connecting the cuneus and mesial temporal regions (Latini et al. 2017).

#### The anatomo-functional model

Duffau et al. (2014) separated the ILF into the anterior and posterior segments—the latter further subdivided into upper and lower parts, supporting a unidirectional transmission of visual information from the visual cortex to the VOFA (upper fibres) and VWFA (lower fibres). From the posterior inferior temporal area, the anterior ILF bidirectionally transmits semantic information (Duffau et al. 2014).

### Functional considerations of the ILF

An integral component of the ventral/semantic language network is the connection between the visual cortex and regions that assign meaning to visual stimuli. This is a similar concept to Milner and Goodale’s ventral or “what” stream, but specific to recognition of written words. The ILF subserves this function as involvement in reading has been extensively reported, particularly with respect to the posterior segment (Mandonnet et al. 2007; Duffau et al. 2014; Zemmoura et al. 2015). This function could be mediated through connections with the occipital cortex and the heavily debated VWFA. In contrast, the anterior ILF segment, hypothesized to be connecting anterior to posterior regions of the temporal lobe, has been associated with semantic functioning (Mehta et al. 2016; Herbet et al. 2016).

### Clinical functional implications: evidence from DES

#### Reading

DES was critical in establishing ILF involvement in visual object recognition and reading. In a seven-patient surgical series using the MT86 reading test, a comprehensive selection of word stimuli, DES of the anterior ILF did not result in reading deficits, but DES of the posterior VWFA produced deficits in reading regular, irregular, and pseudowords, a combination of symptoms termed “complete alexia” (Zemmoura et al. 2015). In the same series, phonological (i.e. irregular word reading) deficits occurred during DES of the anterior VWFA (Zemmoura et al. 2015).

#### Naming and visual agnosia

In contrast to reading, DES of the ILF does not appear to consistently result in visual object-naming deficits, possibly due to IFOF compensation. In a surgical series of 12 patients harbouring low-grade glioma, no deficits were recorded on the DO-80 picture-naming task (Mandonnet et al. 2007). The hodotopical language model supports object and word recognition by the upper and lower ILF fibres, respectively, with stimulation resulting in anomia for the upper fibres and alexia for the lower fibres (Duffau et al. 2014). These impairments in naming could be a result of transient semantic stream deficits in accessing information related to visually presented objects. This is supported by reports of visual hemi-agnosia upon stimulating the ILF bilaterally, but using small (1–3 patient) sample sizes (Coello et al. 2013; Mandonnet et al. 2009; Gil-Robles et al. 2013).

### Clinical functional implications: evidence from disconnection syndromes

#### Reading

Disrupting the visual cortex–posterior ILF–VWFA connectivity results in alexia (Zemmoura et al. 2015). Analysis of postoperative resection cavities in seven patients with MRI and dMRI showed that although resection of the anterior ILF led to only transient disruptions in reading, posterior ILF disconnection led to pure alexia or alexia without aphasia or agraphia, as well as impaired reading of regular, irregular, and pseudowords (Zemmoura et al. 2015), indicative of a word-processing deficit in early stages of word form recognition. In patients with ILF resections, complete recovery of reading difficulties has been observed in lesions anterior to the posterior inferior temporal cortex (Zemmoura et al. 2015), which is the hypothesized location of the VWFA, suggesting participation in reading networks. Further support for the posterior occipital–VWFA connection comes from Zemmoura et al. (2015), reporting lesions posterior to the VWFA lead to alexia.

Literature from dyslexia supports resection observations, suggesting ILF participation in orthographic processing (Vandermosten et al. 2012a). Orthographic processing is an approach to reading that develops after one has built a system of grapheme–phoneme representations in childhood, allowing for the direct relation of a written word to phonological and semantic concepts. This concept is supported surgically; in a lesion-mask subtraction analysis of patients with brain tumours, surface dyslexia, a subset of dyslexia involving over‐reliance on the grapheme–phoneme route with maintained reading of words and pseudowords, was found to involve the ILF (Tomasino et al. 2020).

#### Naming

Damage to the ILF has been associated with naming difficulties, potentially stemming from a necessary role of the tract in lexical retrieval and connections to semantic hubs in the ATL. Specifically, damage to the ILF has been associated with impaired naming for animals, fruits and vegetables, and musical instruments (Mehta et al. 2016). In one surgical series, resecting at least one portion of the anterior ILF resulted in transient postoperative naming difficulties, although resection cavities were extensive, including the temporal pole, anterior superior temporal gyrus, middle temporal gyrus, and anterior fusiform gyrus (Mandonnet et al. 2007). Specifically, a necessary role of the ILF in lexical retrieval has been suggested, as a large voxel-based lesion-symptom mapping study found disconnection of the ILF predicted chronic lexical retrieval impairments (Herbet et al. 2016).

The ILF–ATL connection has been implicated in semantic functioning (Turken and Dronkers 2011), also supported by lexicosemantic impairments from the semantic variant of primary progressive aphasia (PPA) (Marcotte et al. 2017). Diffusion MRI data have also identified radial diffusivity (RD) relation to lexicosemantic deficits in the ILF bilaterally and lexical-richness in the left ILF only (Marcotte et al. 2017).

#### Neuropsychological syndromes

In an analysis of 23 adolescents with schizophrenia or schizoaffective disorder and 21 controls, significantly reduced FA and increased RD in the left ILF was reported (Ashtari et al. 2007). Clinical symptoms were also found to be predictive of left FA, with lower values in patients with history of visual hallucinations (Ashtari et al. 2007). Left ILF reduced FA values were replicated in adults with first-episode (Cheung et al. 2008) and chronic schizophrenia (Liu et al. 2013). Specifically in the right ILF, reduced FA values were correlated with increased thinking disorder scores, a positive symptom (Phillips et al. 2009). The role of the ILF bilaterally in psychosis is also supported by the genetic neurodevelopmental condition, 22q11.2 deletion syndrome (Tylee et al. 2017).

Finally, Boets et al. (2018) found that adolescents with Autism Spectrum Disorder (ASD), a developmental disorder in which visual information related to emotion is commonly affected, had significantly reduced FA values in the right ILF compared to controls. These changes in the right ILF corresponded with slower target detection during visual search and a fragmented part-oriented method of processing of images, although statistically non-significant (*p* = 0.059), possibly due to reduced statistical power from low sample size (Boets et al. 2018).

## Middle longitudinal fasciculus (MLF)

### History of the MLF

The MLF constitutes the most recently discovered ventral language tract identified in 1984 by Seltzer and Pandya (1984) using autoradiography techniques to investigate parieto-temporal tracts in rhesus monkeys originating in the caudal inferior parietal lobe (architectonic area PG/Opt) and terminating in the cortex surrounding the superior temporal sulcus (architectonic area TPO, PGa, and IPa). Data were subsequently replicated in non-human primates using diffusion spectrum imaging (DSI) (Schmahmann et al. 2007).

### Fibre anatomy of the MLF

#### Terminations

**Fig. 3:**
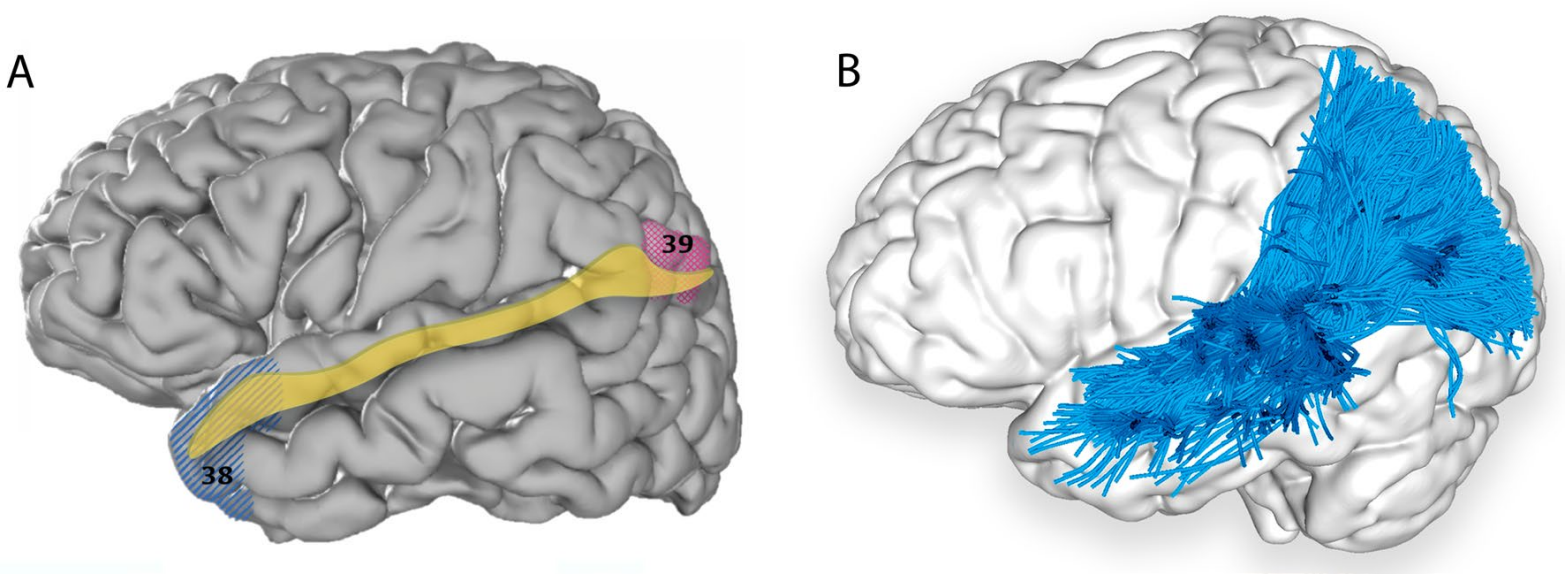
**A** Cortical projection of MLF fibres (yellow) with numbered BAs which interconnect, created with the same methods as in Fig. 1A. **B** Left lateral view of the brain superimposed with the left MLF (red) derived by automated white matter tract parcellation as in Fig. 1D. 20% of the total fibres comprising the tract have been displayed and further refined by using multiple negative regions of interest (ROIs) to be reflective of a morphologically classical representation of the MLF

DSI, dMRI, and microdissection techniques have been employed to identify precise MLF terminations. While initial reports supported the MLF as predominantly connecting anterior to posterior temporal regions, recent imaging has confirmed numerous parietal terminations, consistent with findings from rhesus monkeys. The MLF was identified recently with available comprehensive methodologies and consistent nomenclature, leading to a consensus in its anatomical projections, including the superior parietal lobe (Makris et al. 2013, 2017; Wang et al. 2013; Kalyvas et al. 2020), angular gyrus (Makris et al. 2009, 2013, 2017; De Champfleur et al. 2013; Wang et al. 2013), superior temporal gyrus (Makris et al. 2009, 2013, 2017; De Champfleur et al. 2013; Wang et al. 2013; Kalyvas et al. 2020), and temporal pole (Makris et al. 2009, 2013, 2017; De Champfleur et al. 2013; Wang et al. 2013; Kalyvas et al. 2020) (Fig. 3, Table 1).

### Functional considerations of the MLF

#### Language comprehension

Data from MLF anatomy in rhesus monkeys shows connections to auditory association cortices in the superior temporal gyrus and superior temporal sulcus, suggesting MLF involvement in sound comprehension in primates, which potentially evolved to auditory comprehension of language in humans (Makris et al. 2013). Given the role of the superior temporal gyrus and angular gyrus in auditory sentence comprehension tasks, the MLF appears to participate in the language comprehension network of the dominant hemisphere (Turken and Dronkers 2011). In the dominant hemisphere, MLF may participate in encoding sub-lexical representations into articulatory forms, word production, and acoustic–phonetic word processing (Makris et al. 2013). It has been proposed the MLF is involved in both semantic and phonological processing (Saur et al. 2008, 2010), connecting the dorsal and semantic language streams.

#### Visuospatial role

While in the dominant hemisphere the MLF’s primary role is language processing, in the non-dominant hemisphere additional properties of this tract include visuospatial and attention functions (Makris et al. 2013). Makris et al. (2013) postulate the MLF may integrate visual and auditory functions through connections of the superior parietal lobe, angular gyrus, and superior temporal gyrus.

### Clinical functional implications: DES and disconnection syndromes

#### Transient or no language deficits

Studies employing DES and investigating disconnection syndromes of the MLF are extremely limited. In a series of eight patients undergoing awake surgery involving large segments of the MLF, and despite identifying positive cortical sites, no language deficits were observed during DES and no new permanent deficits were found postoperatively, postulating the MLF is non-essential language tract in humans (De Hamer et al. 2011). In the immediate postoperative period, patients presented primarily with temporary anomia and semantic paraphasia (De Hamer et al. 2011). Based on transient deficits and the large volume of the disease, averaging 62 ml (De Hamer et al. 2011), it is likely the MLF plays a supportive role in semantic processing, but this function can be compensated.

## Inferior fronto-occipital fasciculus (IFOF)

### History of the IFOF

A fronto-occipital fasciculus (FOF) was attributed rather erroneously by Forel and Onufrowicz, while describing aberrant callosal fibres in callosal agenesis patients, but Dejerine identified the error and assigned to the FOF its proper fronto-parietal-occipital connections (Dejerine 1895). In non-human primates the tract was named post hoc, superior FOF, rather imprecisely (Schmahmann and Pandya 2006). However, the IFOF is the only language-relevant, ventral white matter tract absent in non-human primates. Curran dissected the IFOF in 1909, closest to its current understanding (Curran 1909). The tract has been consistently identified with numerous techniques, including fibre dissections, dMRI, and DSI.

### Fibre anatomy and models of the IFOF

#### Frontal terminations

**Fig. 4:**
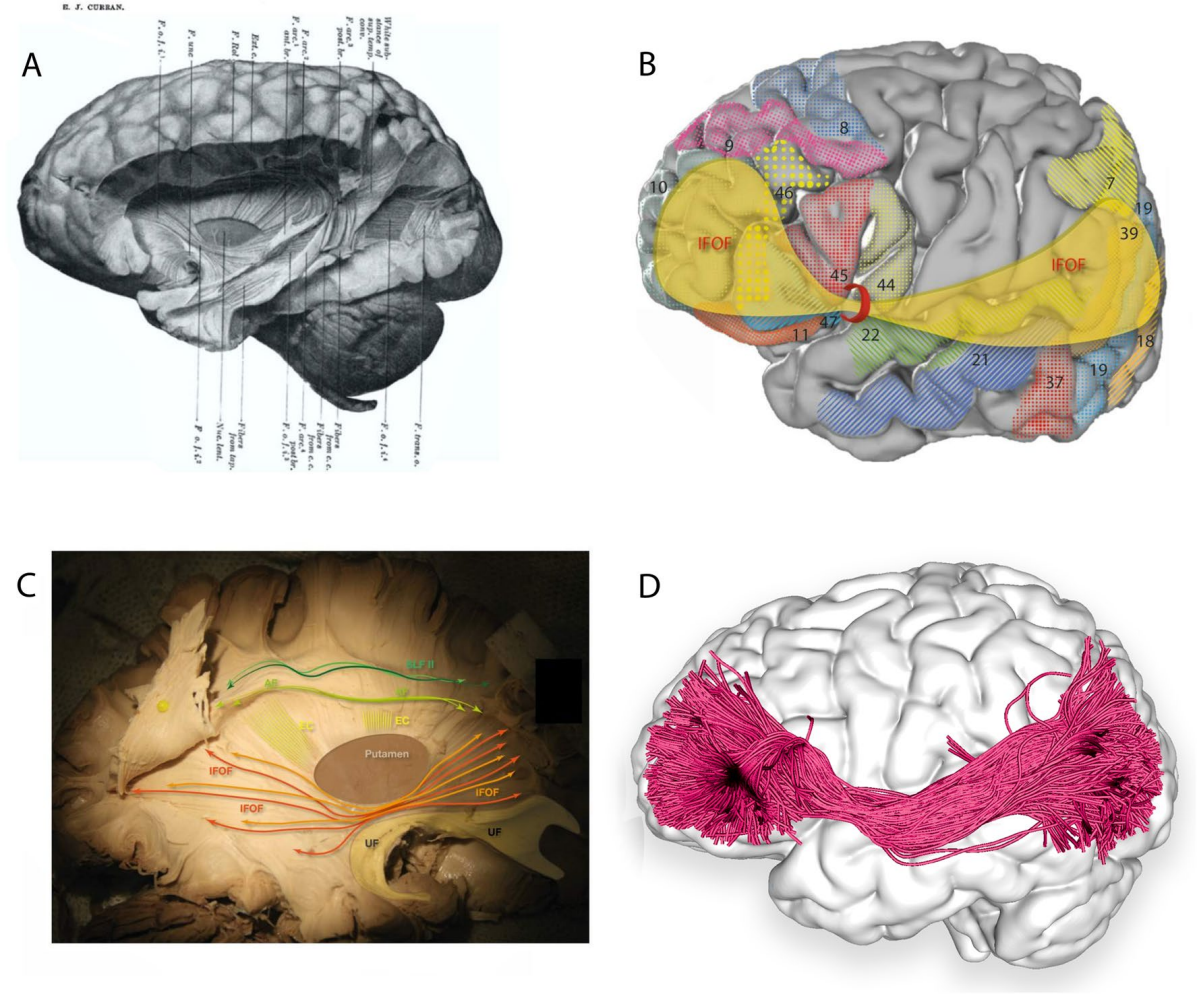
**A** Plate 1, Fig. 1 from 1909 Curran's description of the IFOF (Curran 1909) Copyright © 1909 The Wistar Institute of Anatomy and Biology, **B** cortical projection of IFOF fibres (yellow) with numbered Brodmann areas which interconnect, created with the same methods as Fig. 1A. **C** Original fibre dissection of the right IFOF on a hemisphere prepared with the Klinger technique. **D** Left lateral view of the brain superimposed with the left inferior fronto-occipital fasciculus (green) derived by automated white matter tract parcellation as in Fig. 1C. 30% of the total fibres comprising the tract have been displayed and further refined by using multiple negative regions of interest (ROIs) to be reflective of a morphologically classical representation of the inferior fronto-occipital fasciculus. *AF* arcuate fasciculus, *EC* external capsule (dorsal claustrocortical system), *F.o.f.* inferior fronto-occipital fasciclus, *SLF II* second branch of superior longitudinal fasciculus, *UF* uncinate fasciculus

Despite its name, terminations of the IFOF are extensively described in all four lobes (Fig. 4, Table 1). Frontal terminations are most extensive and include the inferior frontal gyrus (Zhang et al. 2010; Sarubbo et al. 2013; Hau et al. 2016); frontal pole (Thiebaut de Schotten et al. 2012; Sarubbo et al. 2013); middle frontal gyrus (Zhang et al. 2010; Sarubbo et al. 2013; Hau et al. 2016); lateral fronto-orbital gyrus (Lawes et al. 2008; Zhang et al. 2010; Sarubbo et al. 2013; Hau et al. 2016); medial fronto-orbital cortex (Zhang et al. 2010; Thiebaut de Schotten et al. 2012; Hau et al. 2016); and superior frontal gyrus (Zhang et al. 2010; Thiebaut de Schotten et al. 2012; Hau et al. 2016).

#### Occipital terminations

The numerous occipital terminations include the middle occipital (Lawes et al. 2008; Martino et al. 2010; Zhang et al. 2010; Hau et al. 2016); inferior occipital (Lawes et al. 2008; Martino et al. 2010; Zhang et al. 2010; Hau et al. 2016); and lingual gyri (Catani et al. 2002; Lawes et al. 2008; Hau et al. 2016) and the cuneus (Hau et al. 2016).

#### Parietal and temporal terminations

Parietal terminations are less commonly identified and include the angular gyrus (Hau et al. 2016) and superior partial lobe (Martino et al. 2010; Sarubbo et al. 2013; Hau et al. 2016). Temporal regions are also less commonly described and primarily limited to the fusiform area (Catani et al. 2002; Sarubbo et al. 2013; Hau et al. 2016), and possibly the superior (Hau et al. 2016) and middle temporal gyrus (Catani et al. 2002; Hau et al. 2016).

#### The two-layer model

In a 14-patient dissection study, Martino and colleagues report two subcomponents of the IFOF originating from the frontal lobe: a dorsal/superficial segment connecting the frontal lobe with the superior parietal lobe and superior and middle occipital cortex, and a ventral/deep segment connecting the frontal lobe with the posterior basal temporal lobe and inferior occipital cortex (Martino et al. 2010).

#### The four-layer model

Sarubbo et al. (2013), using cadaveric dissection of ten hemispheres and single-subject dMRI, reported superficial and deep cortical-directed IFOF layers. The superficial IFOF was directed antero-superiorly with anterior terminations in the pars triangularis and pars orbitalis, and posterior terminations in the fusiform area, superior parietal lobule, and extra-striate cortex (Sarubbo et al. 2013). The deep IFOF was further dissected into three layers: anterior, projecting from the basal orbitofrontal cortex to the fusiform area and extra-striate cortex; middle, projecting from the middle frontal gyrus/lateral orbitofrontal cortex toward the superior parietal lobe; and posterior, originating in the middle frontal gyrus/dorsolateral prefrontal cortex and terminating in the superior parietal lobule, extra-striate cortex, and fusiform area (Sarubbo et al. 2013).

#### Variability and lateralisation

New evidence from a group performing a stem-based anatomical virtual dissection of the IFOF in a dataset of 60 healthy adults found substantial heterogeneity in IFOF terminations among individuals (Hau et al. 2016). Consistent components (present in the right and left hemispheres of ≥ 50% of subjects) included the inferior and middle frontal gyrus, lateral fronto-orbital gyrus, middle occipital gyrus, and lingual gyrus (Hau et al. 2016). The remaining, less consistent IFOF components were the superior frontal gyrus, medial fronto-orbital gyrus, inferior occipital gyrus, cuneus, superior and middle temporal gyrus, fusiform gyrus, superior parietal gyrus, and angular gyrus (Hau et al. 2016).

The asymmetry index of the IFOF showed right lateralisation for lateral projections, specifically inferior frontal, middle occipital, and inferior occipital projections, whereas medial projections demonstrated leftward lateralisation (i.e. medial fronto-orbital gyrus, lingual gyrus, and cuneus) (Hau et al. 2016). In an investigation of sub-insular microstructure, significant sub-insular leftward FA asymmetry was found (Rodrigo et al. 2007). However, different reports show no asymmetry between the left and right hemispheres (Wu et al. 2016).

## Functional considerations of the IFOF

The IFOF has been consistently implicated in semantic processing and the tract’s extra-striate connectivity (Sarubbo et al. 2013) supports involvement in object discrimination. Given its tract terminations in the frontal lobe, parts of the IFOF could serve as a source of integration between the dorsal and ventral language stream, while other parts could retain an exclusive role in semantic functioning, given the IFOF is implicated in both visual object naming (requiring stating aloud the name of an item) and non-verbal semantic associations. Based on the IFOF’s connections with the inferior frontal gyrus, areas involved in executive functioning aspects of semantic processing, the frontal connections could additionally serve this role (Duffau et al. 2005). Connections between the dorsomedial occipital and parietal areas with the caudo-dorsal prefrontal cortex, areas involved in visuospatial function, the right IFOF could also subserve this modality.

### Clinical functional implications: evidence from DES

#### Semantics and naming

DES reproducibly results in transient semantic paraphasia (Epelbaum et al. 2008; Duffau et al. 2009; De Hamer et al. 2011; Papagno et al. 2011; Zemmoura et al. 2015), with additional language deficits conditional to stimulation sites. Semantic paraphasias may be associative, replacing the target word with a word which is semantically related but not in the same category (e.g. “key” instead of “padlock”) or coordinate, replacing a target word with a word in the same category (e.g. “tiger” instead of “lion”), regardless of the part of IFOF stimulated (Duffau et al. 2002). However, the extent to which associative versus coordinate errors occur is unclear due to low sample size.

Similarly, Moritz-Gasser et al. (2013) supported a multimodal role of the left IFOF in semantics when DES elicited anomia and semantic paraphasia, but also non-verbal semantic associations on the Pyramids and Palm Trees Test (PPTT), a task in which patients are presented with an item (e.g. pyramid) and then instructed to match the item to a target (e.g. palm tree) or distractor (e.g. fir tree) (Epelbaum et al. 2008; Duffau et al. 2013; Moritz-Gasser et al. 2013). It has been suggested that different layers of this tract could have different specialisations in semantic processing. The superficial and deeper components may be involved, separately, in the verbal semantic and non-verbal semantic functions, respectively (Duffau et al. 2013; Moritz-Gasser et al. 2013).

#### Reading and writing

A case report suggested involvement in reading and writing, as transient alexia and agraphia were reported from subcortical DES of its deep parietal terminations (Motomura et al. 2014), supporting Sarubbo’s model (Sarubbo et al. 2013) of posterior and middle portions of the deep IFOF involvement in multimodal integration, regions coinciding with the reading and writing areas identified by Motomura et al. (2014).

### Clinical functional implications: evidence from disconnection syndromes

#### Language and memory deficits

Left IFOF disconnection syndromes result in varied deficits, likely attributed to the IFOF’s broad connections spanning all four lobes. Disconnection syndromes are associated with impaired semantic processing (Han et al. 2013), visual and verbal memory (Bigler et al. 2010), and processing speed (Liu et al. 2013). A significant relationship between performance on verbal memory and visual memory tests with FA and verbal memory with apparent diffusion coefficient (ADC) of the left, but not right IFOF, has been reported (Bigler et al. 2010). A dMRI study in 17 patients with chronic schizophrenia and 17 healthy controls demonstrated left IFOF FA reduction significantly correlated with impaired speed of processing, and verbal and visual learning (Liu et al. 2013).

#### Dyslexia

The transient reading disturbances identified during DES stimulation are consistent with tractography findings from adults with dyslexia, identifying a role for the IFOF in orthographic processing of written words, rather than grapheme–phoneme conversion (Vandermosten et al. 2012a, b). The IFOF is hypothesized to share this role with the ILF, which is consistent with similar posterior projections (occipital and temporal) of these language tracts, which may both run through the VWFA.

#### Non-dominant side

In the right hemisphere, spatial neglect has been reported, with high probability of IFOF disconnection (Herbet et al. 2017). In addition, non-verbal semantic processing and face-based mentalizing, assessed with PPTT and ‘Reading the Mind in the Eyes’ tasks, respectively, identified a temporoparietal junction termination corresponding to both mentalizing and semantic judgement (Yordanova et al. 2017). Lesion-deficit mapping was used to assess the ability to recognize six emotional facial expressions in 103 patients with focal lesions and reported emotion recognition impairments associated with right IFOF damage (Philippi et al. 2009).

#### Neurology/neuropsychology deficits

Kvickström and colleagues (2011) studied the IFOF in progressive supranuclear palsy using mean values of right and left ROIs and found significantly decreased FA and increased ADC in frontal IFOF segments. Bilaterally, the IFOF has also been implicated in antisocial behaviours and Alzheimer’s disease (Smith et al. 2010; Waller et al. 2017). A separate study in 255 children found a positive correlation of right IFOF volumes with the obsessive compulsive disorder (OCD) symptom of doubt-checking (Suñol et al. 2018). Interestingly, in adolescents with OCD, increased FA in the right IFOF has been reported (Zarei et al. 2011), but the opposite trend appears in adults with OCD (i.e. reduced FA values bilaterally) (Garibotto et al. 2010).

## Concluding remarks

**Fig. 5:**
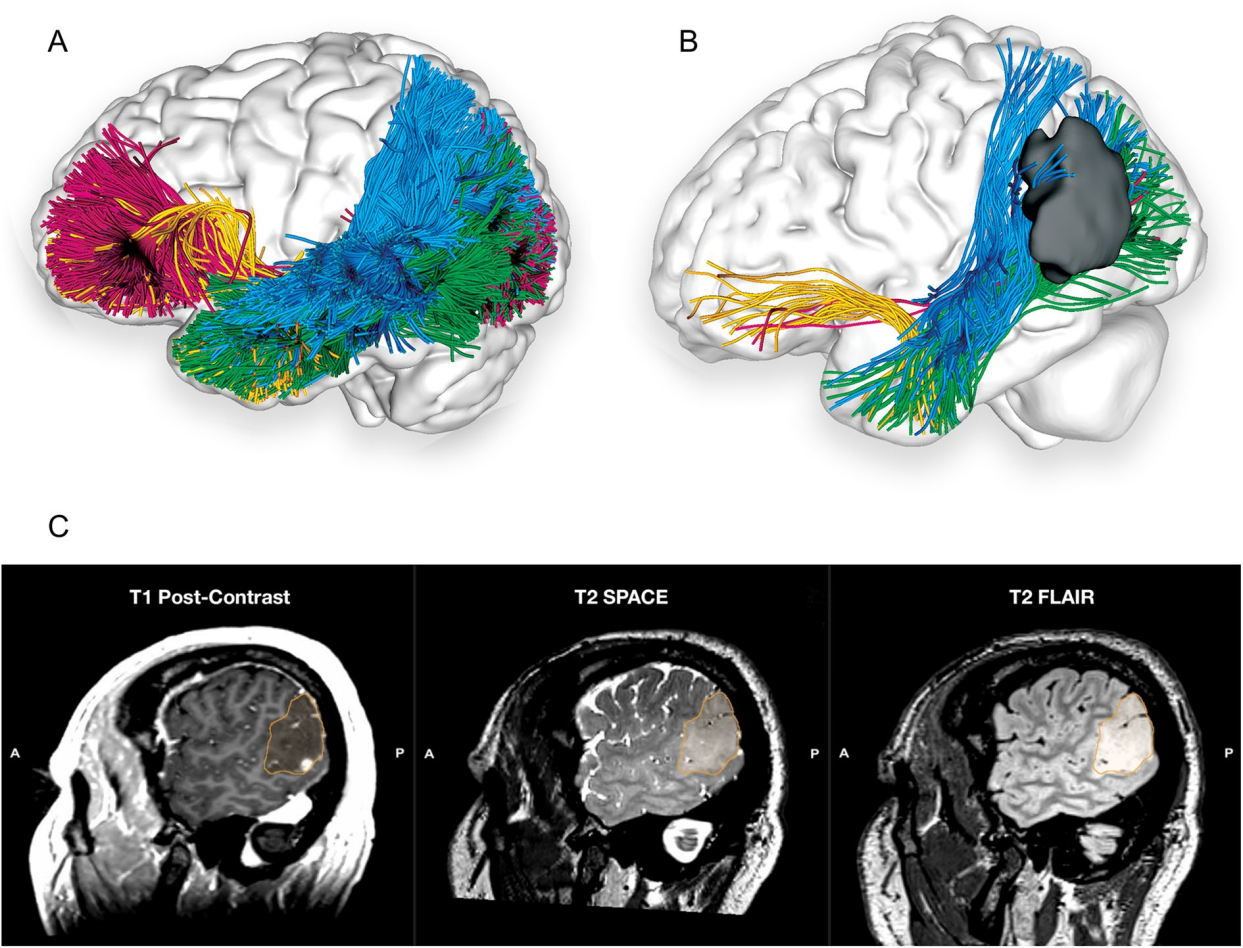
**A** Left lateral view of the brain superimposed with a composite of the tracts from Figs. 1C, 2C, 3B and 4D comprising the ventral verbal processing stream in a single-subject dataset from the Human Connectome Project (Van Essen et al. 2013). **B** Left lateral view of the patient’s brain superimposed with a composite of the tracts comprising the ventral verbal processing stream as described in Fig. 1D. UKF tractography with free water modelling is used to compensate for the decreased FA due to peritumoural oedema (Gong et al. 2018). **C** Co-registered sagittal slices of the T1 post-contrast, T2 SPACE and T2 FLAIR for a patient with a WHO Grade IV IDH-wt left temporoparietal junction glioma infiltrating both the grey and white matter

The current review provides an interdisciplinary, comprehensive, integrative synthesis of data on four key white matter tracts subserving the ventral, semantic language network (Fig. 5). The findings of diverse disciplines were considered, including anatomical, blunt fibre dissection studies; histochemical tract-tracing techniques; termination studies and segmentation models developed using various dMRI methods; functional considerations including findings from neuropsychiatry and clinical neurology syndromes, DES, and lesioning disconnection syndromes.

Although each discipline provides unique dataset inputs, an interdisciplinary data integration analysis is currently lacking. In addition to synthesizing diverse data outputs in a single review, some additional points have been presented, aiming to facilitate further hypothesis testing. For example, the present review suggests that integratory functions between the dorsal (phonological) and ventral (semantic) stream of the UF may be located more anteriorly in the orbitofrontal cortex, and more distributed in the ATL than suggested by the dual-stream model by Hickok and Poeppel (2007), which includes a connection between the posterior inferior temporal gyrus, anterior medial temporal gyrus, and inferior temporal sulcus. The remaining three white matter tracts include more distributed terminations than suggested by the dual-stream model, with the ILF containing fibres spanning the occipital cortex to the temporal pole, the MLF reaching the superior parietal lobule, temporal pole, and occipital cortex, and the IFOF with reported terminations in all four lobes. However, it is unlikely all terminations are involved in semantic processing as DES and disconnection syndrome studies demonstrate.

Our synthesis further identified several gaps in our current understanding of white matter tracts. First, controversy remains regarding the role of the UF in naming with inconsistent outputs from studies despite similar resection cavities; therefore, significant interobserver variability exists. This could be clarified by performing large-scale lesion/symptom analysis in patient cohorts with stroke and interrogating existing stroke databases such as the PLORAS data depository (Price et al. 2010). Second, additional data are required to support the compensatory nature of different ventral tracts (e.g. IFOF compensation for UF or ILF disconnection) and the time intervals required for these pathways to interact during task performance. Here, dMRI and dynamic causal modelling studies may be of use (Seghier et al. 2012). Further identification of alternative pathways and their recruitment after brain lesioning may predict outcomes of patient rehabilitation (Seghier et al. 2012). Third, as the most recently discovered tract of the semantic language network, further studies are required to define the MLF’s additional functions (Makris et al. 2013). Fourth, while IFOF’s semantic function is supported, its role in multimodal processing remains unclear (Sarubbo et al. 2013; Motomura et al. 2014). In addition, inter-subject variability, involving reduced reliance on aggregated statistical measures and associated biases when using different cognitive strategies for a specific task, should be strongly considered (Seghier and Price 2018).

Diverse neuroscience disciplines will greatly benefit from integration and investigation of out-of-specialist datasets, generating further research hypotheses that can be tested with interdisciplinary collaboration. The current review aims to facilitate this direction, in addition to maximizing cognitive function preservation during neurosurgical operations. This is particularly pertinent as recent studies and a meta-analysis have shown that language testing with picture naming dominates ABM during tumour removal (Ruis 2018; Sefcikova et al. 2020). Widening the brain mapping paradigms with region-specific testing, based on the detailed multidisciplinary data discussed above, will not only help preserve a much broader spectrum of high-order cognitive functions, but may help validate cognitive neuroscience models, or proposal new ones.

## Data Availability

Data used to develop images were, in part, collected from the published Human Connectome Project database. Raw data can be retrieved from: https://db.humanconnectome.org.
